# Alius, on the Subject of Quackery

**Published:** 1799-10

**Authors:** 


### *To the Editors of the Medical and Phyfical Journal.*

GENTLEMEN,

HAVING read with pleafure, in your *Journal* for June, fome obfervations on the fubject of Quackery, by ALIQUIS, I have taken the liberty of making a few remarks on the fame fubject, by inferting which in your valuable work, you will oblige

Your moft obedient fervant,

ALIUS.

The fubject which has occupied the attention, and engaged the pen of the writer referred to, is undoubtedly an important one, and the remarks which he has made upon it are juft and pertinent. "That evils of fuch magnitude fhould be fuffered to pafs unnoticed" muft excite wonder in the minds of thofe who are capable of judging concerning the extent of the mifchief. The writer, at the fame time, that he points out the evil to be dreaded, with great propriety repels the infinuation "that the regular profeffors of phyfic are interefted in the diffemination of thefe fpurious noftrums, the fuppreffion of which would leffen the progrefs of difeafe, and of courfe diminifh the number of patients who are ultimately compelled to feek relief from them, for the diforders brought on by quacks, mountebanks, empirics, &c."

The plan for erecting a public board for the examination of every new medicine is undoubtedly judicious, and, if properly executed, might contribute, *in a confiderable degree*, to the prevention of at leaft a part of the mifchief to be dreaded as the confequence of the unlimited indulgence which is at prefent afforded to the venders of thefe pretended remedies. But could this plan be carried into effect, according to the benevolent wifh of your correfpondent, would there not ftill remain a fruitful fource of mifchief to the public ?

The author of thefe remarks makes a diftinction between thofe medicines "the compofition of which is known, and the venders of which are ignorant pretenders to medical knowledge, as being in fituations of life remote from the profeffion of phyfic," and thofe which come forth fanctioned by the name of a phyfician of known abilities and integrity. This diftinction is undoubtedly well-founded, and the value of a medicine compounded under the direction of a man of fcience, muft differ widely from that, the ingredients of which are put together in an unfkilful manner, without any regard to the effect which the different articles may have upon each other, and by a perfon totally ignorant of the effects which the compound may produce on the conftitution of the perfon who makes ufe of it. The latter may prove in every cafe inefficacious, and in fome cafes may be injurious, whereas the former when adminiftered with judgment and defign, with a proper regard to the known effects of its different ingredients, and the fuitablenefs of thefe to the removal of the fymptoms, under which the patient labours, may prove a valuable medicine. But let us fuppofe that a method could be adopted to afcertain the ingredients of the medicines referred to, and that hereby the fale of " poifon" in a thoufand forms fhould be prevented—let us farther fuppofe that it fhould appear upon examination that they have been compounded with the greateft care and attention, according to the prefcription of a moft able phyfician, and that they are recommended to be ufed only in fuch complaints as they were originally defigned to relieve—ftill but a partial removal of the evil of which the writer complains, would be obtained. It muft be obvious not only to every man of *medical fcience*, but alfo to every man of *common fenfe*, who thinks clofely on the fubject, that a medicine which has proved effectual for the removal of fome difeafe, may be very improper for a patient labouring under a difeafe bearing the fame name. What would be proper in a particular *ftage* of any difeafe, and under particular *circumftances* which may occur, would be highly improper and might prove injurious, or even fatal, at a different period, or under different circumftances; and it is particularly important to remark, that the more powerful and efficacious the medicine, and the more juft and true the report which which is circulated of its ſurpriſing and ſudden effect in curing diſeaſe, the more dangerous does the uſe of it become, if the application of it in any inſtance ſhould be improper. The medical practitioner who, with great judgment orders a medicine to be taken *to-day,* may ſee it neceſſary to forbid the uſe of it *to-morrow*: it may have anſwered the purpoſe for which it was deſigned; but, that being accompliſhed, the repetition of it may be unneceſſary, or may even be attended with inconvenience. It is not to the uſe of any ſpecific medicine that the cure of a diſeaſe is to be attributed, but to a diligent and careful attention of the practitioner to the different ſymptoms which occur, and to a *change* of medicine, and variation of plan, according to the urgency of theſe ſymptoms, and the alterations taking place in the ſyſtem in conſequence of them.

That an individual, however, who with great labour and ingenuity had diſcovered ſome medical compound, ſhould not fail to reap the advantage of ſuch diſcovery, nor the public be deprived of what, upon proper examination, might prove a valuable remedy, let ſuch a board as the writer propoſes be inſtituted, and upon a report from this board, let the inventor receive from the public ſome valuable compenſation for the diſcovery, or let him continue to be the ſole proprietor and vender; but let it be ſold ONLY to thoſe who, knowing the effects which it is likely to produce, are the proper and the only judges of the inſtances in which it may be employed with ſafety and advantage.

Let the Apothecaries' Company, from time to time, purchaſe of the proprietor ſuch a quantity as they may find neceſſary for their demand.— Let a quantity of it be found in the drawer or in the phial of every private apothecary, ready for uſe, when it may be directed as the whole, or the part of a medical preſcription. In this way let the inventor receive a compenſation for his diligence and ingenuity, and the public be ſecured from the dangerous effects of a compound, which, if it poſſeſs any powers, muſt, in many inſtances, though perhaps in a ſlow and ſecret manner, prove injurious to thoſe who have made uſe of it.

